# Impact of Apical Periodontitis and Non-surgical Root Canal Treatment on Serum Inflammatory Biomarkers in Patients With Cardiovascular Disease With Apical Periodontitis: A Prospective Interventional Study

**DOI:** 10.7759/cureus.87723

**Published:** 2025-07-11

**Authors:** Sushma Bhardwaj, Sanjay Tewari, Kuldip Laller, Ashwani Kumar, Mayank Arora

**Affiliations:** 1 Conservative Dentistry and Endodontics, Pandit Bhagwat Dayal Sharma Post Graduate Institute of Dental Sciences, Rohtak, IND; 2 Cardiology, Pandit Bhagwat Dayal Sharma Post Graduate Institute of Medical Sciences, Rohtak, IND

**Keywords:** apical periodontitis, biomarkers, cardiovascular disease, coronary artery disease, risk factors

## Abstract

Objective

This study's objective was to determine the impact of apical periodontitis (AP) and non-surgical root canal treatment (NSRCT) on the levels of serum high-sensitivity C-reactive protein (hs-CRP) and cardiovascular risk in patients with cardiovascular disease (CVD).

Materials and methods

In this prospective interventional study, 35 patients with coronary artery disease (CAD) with AP and 35 age and gender-matched CAD without AP controls were included. In both groups, serum high-sensitivity C-reactive protein (hs-CRP) and complete hemogram (CH) indices were assessed at baseline. Root canal treatment was done in teeth with AP, and biomarkers were reassessed at 6- and 12-month follow-ups. Mann-Whitney and Wilcoxon signed-rank tests were applied for intergroup and intragroup comparison, respectively. Multiple regression models were used to predict the effect of AP on change in hs-CRP levels after NSRCT after adjusting for possible demographic oral and classic cardiovascular confounders.

Results

A significant difference in hs-CRP levels was observed between patients with CAD with AP [1.95 (0.13-6.34) (2.17)] and controls [1.04 (0.40-3.12) (1.30)] mg/L at baseline. At 12 months post-treatment, there was a significant reduction in hs-CRP levels to 0.55 (0.40-2.4) (0.75) mg/L, and a reduction was also observed in a number of patients classified as a high-risk group with hs-CRP levels >3 mg/L, from 26% to 0%.

Conclusion

Significantly higher systemic inflammatory burden (SIB) as assessed by systemic inflammatory biomarkers was observed in patients with CAD with AP than CAD without AP controls, which reduced significantly after NSRCT, highlighting the efficacy of non-surgical root canal treatment (NSRCT) in reducing SIB and hence, cardiovascular risk.

Clinical relevance

This is the first prospective interventional study in CVD patients to assess the impact of apical periodontitis and non-surgical root canal treatment on systemic inflammatory burden and cardiovascular risk.

## Introduction

Cardiovascular diseases (CVDs) encompass a wide range of conditions affecting the heart and blood vessels, including coronary artery disease (CAD), cerebrovascular disease (stroke), peripheral artery disease (PAD), hypertension, and aortic diseases [1].

Coronary artery disease (CAD) is one of the most common types of CVD and is related to an elevated risk of disability and morbidity within the South Asian demographic [2]. The highest CAD rates are found in Indians [3]. Furthermore, the projected loss of productive years of life due to CAD in India is expected to rise from 7.1 million in 2004 to 17.9 million by 2030 [4]. The pathophysiology of CAD is characterized by atherosclerotic alterations in the coronary artery walls, which impede normal blood flow to the cardiac muscle, resulting in myocardial ischemia and, in severe instances, myocardial infarction and death [5].

Apical periodontitis (AP) is an inflammatory process that occurs because of root canal infection around the apex of the root. At the individual level, the prevalence of AP is 52% and 5% in terms of teeth [6]. A study concluded that the prevalence of AP was significantly higher in individuals with CAD than in the systemically healthy individuals, 50.8%/25.0% [7]. Although AP is considered a local pathological disorder, there is growing evidence implying that AP may lead to increased systemic inflammation [8]. AP is associated with significantly higher values of biomarkers of systemic inflammation, including high-sensitivity C-reactive protein (hs-CRP) [9,10], interleukin-6 (IL-6) [11], etc.

AP-related low-grade systemic inflammation and bacteraemia may have detrimental effects on systemic health, including the progression of CVDs [12]. A recent umbrella review (UR) [13] included four systematic reviews (SR) with 46 studies attempting to assess whether there is a relationship between CVDs and AP. This UR found a weak association between CVD and AP. A latest SR and meta-analysis (MA) [12] pointed to a weak link between AP and CVD in pooled data of eight cross-sectional (CS) studies [combined odds ratio (OR)-1.53], while five case-control (CC) studies (OR-1.24) and two cohort studies (pooled risk ratio-1.27) didn’t show any significant association. Though an association has been observed between AP and CVD in previous observation studies [12,13], no intervention study has been conducted to date in CVD patients.

The reduction in biomarkers after non-surgical root canal treatment (NSRCT) has been observed in several intervention studies in systemically healthy individuals with AP [10,14-20]. The American Heart Association (AHA) and Centers for Disease Control and Prevention (CDC) jointly released guidelines, designating hs-CRP as the preferred inflammatory marker for determining cardiovascular risk and classified hs-CRP levels <1 mg/L as low, 1-3 mg/L as intermediate, and >3 mg/L as a high-risk group for CVD worldwide [21]. Recently, monocyte lymphocyte ratio (MLR), neutrophil-lymphocyte ratio (NLR), total leucocyte count (TLC), platelet lymphocyte ratio (PLR), novel marker systemic immune-inflammation index (SII) and inflammatory cytokines have been investigated to estimate atherosclerotic changes and cardiovascular risk [22]. By routine whole blood count test (a clinical test which is used most), NLR, MLR, PLR, TLC and SII (NLR × platelet count) can be easily calculated and these are more cost-effective than inflammatory cytokines [22].

Despite numerous observational studies identifying an association between oral disease burden and systemic inflammatory markers in the blood of systemically healthy individuals, there are no studies that have yet investigated the effect of AP and NSRCT on systemic inflammatory markers in CVD patients. So, the objective of this prospective study was to evaluate the impact of AP and NSRCT on the levels of hs-CRP and complete hemogram (CH) indices and, hence, the assessment of CVD risk in CAD patients.

## Materials and methods

This prospective interventional study was conducted after ethical approval by the Institutional Ethical Committee (PGIDS/BHRC/22/52). All participants signed the informed consent and were explained the procedure with associated benefits and risks thoroughly.

Patients’ enrollment

Out of 1134 patients who attended the OPD of the cardiology department for cardiac check-ups, 70 patients fulfilling the inclusion criteria were enrolled to participate in this study between October 2022 and March 2023. A total of 70 patients - 35 patients with the diagnosis of CAD with AP (21 molars, 14 premolars, and 7 anterior) and 35 patients with CAD without AP reported - were taken as the test and control group, respectively. Timely treatment of patients is of ethical importance, so CAD with AP patients with delayed treatment was not taken as the control group.

Inclusion criteria were individuals between 40 and 75 years old, the presence of at least eight teeth, diagnosis of CAD as described by the Brazilian Society of Cardiology [23], with proved evidence of either one or more of the following cardiovascular events six months before participating in the study: stable angina and history of myocardial infarction (MI); as evaluated by angiography, more than 50% lesion size in at least one major coronary artery; surgical or percutaneous myocardial revascularization; positive results of non-invasive testing of ischemia and presence of angina [24], AP with periapical index (PAI) scores ≥3 [25] in at least one permanent tooth.

Exclusion criteria were individuals diagnosed with non-communicable diseases other than CAD, obesity with body mass index (BMI) ≥30 kg/m^2^, acute infections, pregnancy, and those on antibiotic and/or anti-inflammatory drugs in the last three months before the pre- and post-treatment follow-up visits and with ≥ stage 2 periodontitis with clinical attachment loss ≥3-4 mm, and maximum probing depth ≥5 mm [26]. Patients who failed to follow oral hygiene instructions and developed new caries were also excluded from the study. Patients were also excluded if there were any changes in the systemic medication regimen (drugs used for treatment of CAD were nitrates, antiplatelet drugs, β blockers, ACE inhibitors and statins in patients’ records).

**Figure 1:**
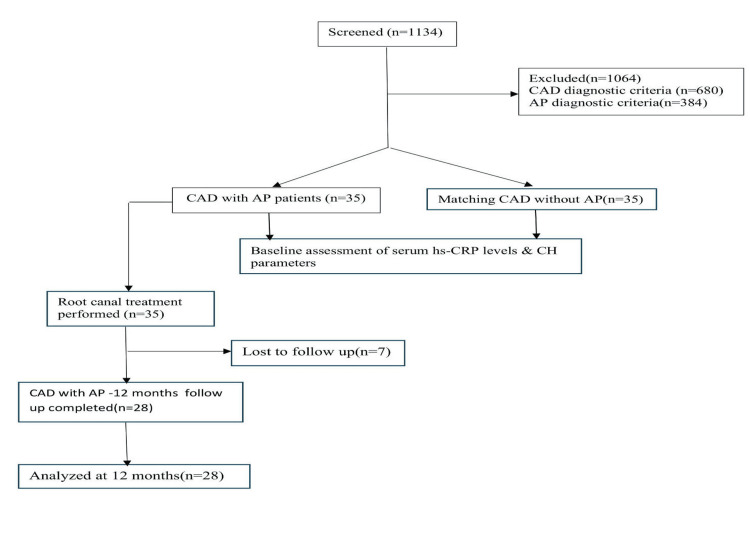
A flowchart of the study design CAD: coronary artery disease; AP: apical periodontitis; CH: complete hemogram

The study flowchart is illustrated in Figure 1.

Sample size determination

Sample size calculation was done using the Fleiss method of the Open Source Epidemiologic Statistics for Public Health (OpenEpi) software (version 3.01, Emory University, Atlanta, USA). Based on a previous study [10] with mean levels of hs-CRP at baseline 3.37 and SD 2.69 in teeth with AP and after NSRCT 1.79 and SD 1.65, using 80% study power, a significance level of 0.05, the sample size was calculated 35 in CAD with AP (Test group) and 35 in CAD without AP (Control group).

Clinical examination

Medical examinations and tests were done to exclude other systemic diseases, such as liver diseases, chronic renal failure, type 1 or type 2 DM, and acute viral infections by liver failure tests (LFT), renal failure tests (RFT), random blood sugar (RBS), and viral marker tests, respectively. Demographic variables and history of CVD and participants' weight, height, and waist circumference were recorded. Two readings of patients’ blood pressure were taken, and an average was recorded. Patients recruited underwent intraoral examinations, and periapical radiographs were taken using standard exposure parameters to assess the intraoral status of suspected teeth with AP with either symptoms of pain, discoloration, dental caries, or non-vital teeth at baseline and at 12-month follow-up in both groups. Periodontal diagnosis was made using clinical parameters, including probing pocket depths, bleeding on probing, and clinical attachment levels, at the base of the gingival crevice. All teeth, except third molars, were assessed at six sites. For the periodontal assessment, a manual periodontal probe (UNC 15; Hu-Friedy, Chicago, IL, USA) was used. Oral prophylaxis was done two weeks before NSRCT. Patients were advised to follow oral hygiene instructions, and those patients not maintaining proper oral health, as assessed by the modified oral hygiene index, were dropped from the study. Clinical examinations, percussion, cold and electric pulp sensibility tests were performed on teeth suspected of presenting AP.

Laboratory analysis

Fasting samples were collected from participants’ blood by venipuncture of the antecubital vein before treatment, at six months, and 12 months in the test group and at baseline in the control group, after two weeks of oral prophylaxis. Fractions of blood samples were tested for lipid profiles (TC-Total cholesterol, TGs-Triglycerides, LDL-Low density lipoproteins, VLDL-Very low-density lipoproteins, etc.), RBS, LFT, RFT, and viral biomarkers. Additionally, the blood sample fraction was drawn into two distinct vacuum blood collection tubes: one with ethylenediaminetetraacetic acid (EDTA) added for anticoagulation and the second for serum separation without any additive for CH and hs-CRP quantitative analysis, respectively. An automated hematology analyzer was used to analyze CH. Assessment of hs-CRP was done by immunoturbidimetry.

Non-surgical root canal treatment (NSRCT)

A single operator performed non-surgical root canal treatment. Under rubber dam isolation, access cavity preparation was done after the local anaesthesia (LA) application. Root canal orifices exploration and negotiation were done with #10 and #15 K files. Using an apex locator (Root ZX apex locator, J. Morita Corporation, Kyoto, Japan), the working length was determined along with radiographs for its confirmation. Rotary endodontic files (Neoendo files, Orikam Healthcare, Gurugram, India) were used for the crown-down technique to perform root canal preparation. Intermittently, irrigation was done with 5.25% sodium hypochlorite (Septodont Healthcare, Navi Mumbai, India) using 27 G side-vented needles, and root canals were obturated with gutta-percha (Meta Biomed Co., South Korea) and zinc oxide eugenol sealer using the lateral condensation method. Composite resin (3M, Saint Paul, MN, USA) was used for the final coronal restoration.

Treatment outcome

Hs-CRP and CH indices were assessed in both groups at baseline, and in the test group, follow-up was done at six months and 12 months as the primary outcome. PAI values were used to measure the radiographic outcome of NSRCT using the five-point scoring system [25] at 0, 6, and 12 months. Lesion size was measured by using CorelDRAW software (Alludo, Ottawa, Ontario, Canada). Further dichotomization of PAI scores was done as healed (PAI ≤2) and unhealed (PAI ≥2).

Statistical analysis

Data was analyzed using SPSS version 22.0 software (IBM Corp., Armonk, NY, USA). Normality test (Shapiro-Wilk test) was performed to check the normalcy of data. Mean ± standard deviation (SD) and median (minimum-maximum) (interquartile range [IQR]) were used to present descriptive statistics. For the data that was not normally distributed, non-parametric tests were applied to compare the levels of inflammatory biomarkers between two groups at baseline using the Mann-Whitney test and within the group at 0, 6 and 12 months, using the Wilcoxon signed-rank test. Correlation of change in PAI score from baseline to 12 months with change in hs-CRP levels from baseline to 12 months was tested using Spearman correlation. To estimate the effect of change in PAI score from baseline to 12 months on the change in hs-CRP levels from baseline to 12 months after adjusting for potential confounders like age, gender, BMI, serum total cholesterol (TC), LDL, HDL, VLDL, and statins at baseline, multiple linear regression models were applied using the enter method. p<0.05 was set as the level of significance.

## Results

A total of 70 patients (60 males and 10 females) - 35 patients in CAD with AP group and 35 patients in CAD without AP group - were analyzed at baseline. At six and 12 months follow-up, 28 CAD with AP patients were finally included for interpretation of results (three patients relocated and were ‘dropouts’, one patient didn’t maintain oral hygiene, one patient had very high hs-CRP suggestive of any unknown acute infection and two patients had change in drug regimen before six months follow-up).

Demographic data of patients is listed in Table 1. No significant difference was observed in age, gender, and BMI between the groups (p > 0.05). Significantly higher values of hs-CRP, LDL, and TC were reported in the group with CAD with AP compared to the group with CAD alone (p-value <0.05). SII and PLR levels were higher at baseline in CAD with AP group, though the levels were not significantly high.

**Table 1:** Demographic data and comparison of inflammatory biomarkers at baseline in CAD with AP (Test) and CAD without AP (Control) groups. BMI: Body mass index, RBS: Random blood sugar, VLDL: Very low-density lipoprotein, LDL: Low-density lipoprotein, HDL: High-density lipoprotein, hs-CRP: high sensitivity C-reactive protein, NLR: Neutrophil-lymphocyte ratio, MLR: Monocyte lymphocyte ratio, TLC: Total leucocyte count, PLR: Platelet lymphocyte ratio, SII: Systemic immune inflammation index. Note- **signifies significant differences between the two groups (p-value <0.05). Values are presented as (Mean±SD) and Median, Minimum-Maximum, Interquartile Range (IQR), except for gender, which is the number (percentage) of patients.

BASELINE CHARACTERISTICS		TEST GROUP (n=35)				CONTROL GROUP (n=35)				p-value
		Mean±SD	Median	Minimum-maximum	IQR	Mean±SD	Median	Minimum-maximum	IQR	
Age (Years)		59.34±9.406	60	42-75	12	58.63±9.069	60	42-75	15	0.747
Gender	Male	31 (88.5)				31 (88.5)				1
	Female	4 (11.5)				4 (11.5)				
BMI (Kg/m^2^)		23.28±1.125	23.5	21.2-24.9	1.5	23.314±1.060	23.5	21.2-24.9	6	0.981
Waist circumference (cm)		88.63±3.50	88	76-89	3	88.26±4.017	88	75-89	4	0.833
RBS (mg/dl)		102.77±16.52	103	65-139	28	105.17±14.69	101	80-130	24	0.664
Total cholesterol (mg/dl)		168.47±50.21	161	100-287	72	145.45±39.69	140	100-237	52	0.034**
LDL (mg/dl)		93.31±38.91	83	35-196	53	76.62±30.46	67	35-147	54	0.042**
HDL (mg/dl)		44.61±10.01	45	27-63	18	39.48±10.45	34	25-63	14	0.026**
Triglycerides (mg/dl)		161.44±80.66	151	77.5-442	114	146.82±67.64	116	80-382	77	0.593
VLDL (mg/dl)		32.38±16.01	30	15.5-88	22	29.51±13.58	23	16-77	16	0.592
Hs-CRP (mg/L)		2.12±1.46	1.95	0.13-6.34	2.17	1.37±0.85	1.04	0.40-3.12	1.30	0.03**
NLR		2.13±1.10	1.766	1.04-6.46	1.07	2.05±1.33	1.7	0.85-8.5	1.16	0.522
TLC Count(/microliters)		7802±2345	7400	4800-18000	1900	8077±2627	8000	5000-14300	3800	0.855
PLR		82.81±29.05	81.25	17.18-147.25	44.41	72.97±44.12	59.36	15.11-182.53	58.86	0.052
MLR		0.238±0.094	0.22	0.06-0.47	0.08	0.196±0.126	0.20	0.02-0.65	0.16	0.086
SII INDEX*10^9 ^(Cells/litre)		375.80±194.17	322.67	61.46-969.23	207.39	331.56±185.53	273.54	61.46-736.67	246.67	0.245

A significant reduction was observed in hs-CRP levels in CAD with AP group at six and 12 months after NSRCT (p-value <0.05). Also, reduction in SII, MLR and PLR after six months of NSRCT was statistically significant in CAD with AP group patients, though no significant reduction was observed at 12 months. The mean PAI score and lesion size were significantly reduced at six and 12 months follow-up after NSRCT (p-value <0.05) (Table 2).

**Table 2:** Lesion size and periapical status, and serum inflammatory markers of patients with CAD with AP at baseline and follow-ups after root canal treatment. hs-CRP: high sensitivity C-reactive protein, NLR: Neutrophil-lymphocyte ratio, MLR: Monocyte lymphocyte ratio, TLC: Total leucocyte count, PLR: Platelet lymphocyte ratio, SII: Systemic immune inflammation index, PAI: Periapical index. Note- p > .05 for follow-up comparison versus baseline, except for **p < .05, and *p = .05 in comparison with baseline. Values are presented as (Mean ± SD) and Median, Minimum-Maximum (min.-max.), Interquartile Range (IQR).

PARAMETERS (Lab & Radiographical)	BASELINE (n=35)				6 MONTHS (n=28)				12 MONTHS (n=28)			
	Mean±SD	Median	Mini.- Maxi.	IQR	Mean±SD	Median	Mini.- Maxi.	IQR	Mean±SD	Median	Mini.- Maxi.	IQR
hs-CRP (mg/L)	2.12±1.46	1.95	0.13-6.34	2.17	1.14±1.11**	0.73**	0.40-4.74**	0.728**	0.82±0.59**	0.55**	0.40-2.4**	0.75**
NLR	2.13±1.10	1.766	1.04-6.46	1.07	2.04±0.619	2.12	0.97-3.09	0.91	2.04±0.749	2.02	0.82-3.5	1.43
TLC Count(/microliters)	7802±2345	7400	4800-18000	1900	7557±1707	7250	4900-11400	2300	6635±1173**	6600**	4000-9100**	1675**
PLR	82.81±29.05	81.25	17.18-147.25	44.41	65.9±27.87**	61.2**	27.05-124.44**	42.2**	2.04±0.75	74	16.65-235.75	61.2
MLR	0.238±0.094	0.22	0.06-0.47	0.08	0.29±0.098**	0.29**	0.1-0.61**	0.09**	0.27±0.11	0.26	0.08-0.65	0.101
SII INDEX*10^9 ^(Cells/litre)	375.80±194.17	322.67	61.46- 969.23	207.39	303.5±165.8**	283.3**	96.78-836.28**	232.95**	334.9±265.5	278.9	61.46-1218.3	266
PAI Score	3.43±0.502	3	3-4	1	2.04±0.51**	2**	1-3**	0**	1.93±0.604**	2**	1-3**	0**
LESION SIZE (mm²)	20.89±10.501	20	3-51	12	6.5±2.58**	6**	2-13**	3**	4.61±2.89**	4**	0-13**	3**

CAD patients with hs-CRP score >3 are considered to be at high cardiovascular risk. In CAD with AP group, 26% had hs-CRP score >3 and in controls CAD without AP group 3% had hs-CRP score >3. Significantly higher values were observed in CAD with AP group than control group (p-value <0.05). After 12 months of NSRCT, hs-CRP level >3 reduced significantly from 26% (before treatment) to 0% (12 months post treatment) in CAD with AP group (p-value <0.05) (Table 3).

**Table 3:** The proportion of subjects classified in three categories of risk for future cardiovascular events based on the level of serum high-sensitivity C-reactive protein in both Groups at baseline and after 12 months of non-surgical root canal treatment in CAD with AP group. Note- ** signifies significant differences (p-value <0.05).

Groups	No risk (<1 mg/L) %(n)	Intermediate risk (1–3 mg/L) %(n)	High-risk (>3 mg/L) %(n)	p-value	Significance
At Baseline					
CAD with AP-35 (Before Treatment)-Baseline	26% (9)	48% (17)	26% (9)	0.015**	Significant
CAD without AP-35 (Control)-Baseline	46% (16)	51% (18)	3% (1)		
After Treatment					
CAD with AP-35 (Baseline) (Before Treatment)	26% (9)	48% (17)	26% (9)	0.000**	Significant
CAD with AP-28 (12 months after treatment)	71% (20)	29% (8)	0% (0)		

Change in hs-CRP from baseline to 12 months was significantly correlated with change in PAI from baseline to 12 months. On multiple linear regression analysis after adjusting the data of other CVD predictors including age, gender, BMI, waist circumference, TGs, LDL, VLDL, TC, and statins at baseline, the change in PAI score was still significant for change in levels of hs-CRP (p-value <0.05) (Table 4).

**Table 4:** Multiple linear regression applied to assess effect of change in PAI score on change in hs-CRP after adjusting data of other CVD predictors including age, gender, BMI, serum cholesterol, HDL, LDL, VLDL and statins (at baseline). Note- ** signifies significant differences (p-value <0.05). BMI: Body mass index, VLDL: Very low density lipoprotein, LDL: Low-density lipoprotein, HDL: High-density lipoprotein, hs-CRP: high sensitivity C-reactive protein, PAI: Periapical index.

Model	Unstandardized Coefficients		Standardized Coefficients	t	Sig.	95.0% Confidence Interval for B	
	B	Std. Error	Beta			Lower Bound	Upper Bound
(Constant)	-6.973	5.862	Not available	-1.190	0.250	-19.290	5.343
Age (years)	0.005	0.022	0.043	0.238	0.814	-0.041	0.051
Gender	1.706	0.712	0.491	2.396	0.028**	0.210	3.203
BMI (Kg/m²)	0.167	0.205	0.161	0.812	0.428	-0.265	0.598
Serum Cholesterol (mg/dl)	-0.001	0.017	-0.033	-0.046	0.964	-0.038	0.036
HDL (mg/dl)	0.041	0.033	0.334	1.255	0.225	-0.028	0.110
LDL (mg/dl)	-0.005	0.017	-0.155	-0.287	0.778	-0.040	0.031
VLDL (mg/dl)	0.010	0.025	0.128	0.392	0.699	-0.042	0.062
Statins- at baseline	-0.795	0.478	-0.326	-1.665	0.113	-1.799	0.208
Change in PAI 0-12	0.740	0.311	0.437	2.380	0.029**	0.087	1.393
Dependent Variable: Change in hs-CRP (0-12) (mg/dl)							

## Discussion

Coronary artery disease (CAD) is a type of CVD that occurs because of coronary arteries occlusion by atherosclerosis and is manifested by stable/unstable angina, MI, or sudden cardiac death [5]. Observational and interventional studies that evaluated the association between AP and CVD by estimation of systemic inflammatory biomarkers have so far been conducted in systemically healthy individuals. The effect of NSRCT on the biomarkers of atherosclerosis in patients already diagnosed with CVD has not been investigated yet. Hence, this study aimed to explore if NSRCT of teeth with AP could be considered a part of preventive strategies for secondary prevention by reduction of systemic inflammation to prevent recurrence of major adverse cardiovascular events in CVD patients.

This prospective study showed that CAD with AP patients have significantly higher levels of hs-CRP than CAD without AP controls at baseline, suggesting that AP contributed to increasing serum hs-CRP levels significantly in CAD patients. These results are consistent with previous studies on systemically healthy individuals reporting significantly raised levels of systemic inflammatory biomarkers in individuals with AP [9,10,16]. Statistically significant reduction in hs-CRP levels after NSRCT was observed at six and 12 months follow-up in the test group. These results are consistent with some previous studies in systemically healthy individuals reporting that NSRCT imparts a reduction in levels of systemic biomarkers [10,15,16,19]. Significantly higher levels of hs-CRP in CAD patients with AP and their significant reduction after NSRCT suggest a cause-and-effect relationship between AP and systemic inflammation.

Further, the number of high-risk hs-CRP patients in CAD with AP group with hs-CRP score >3 reduced significantly from baseline (26%) to 12 months (0%) after NSRCT, emphasizing the necessity of screening of CVD patients for AP and prompt NSRCT of teeth with AP to eliminate the cause of chronic systemic inflammatory burden. This suggested that AP could contribute to raised levels of hs-CRP, and NSRCT could possibly alter the risk of atherosclerosis in stable CAD patients with AP.

In our study, hs-CRP assessment was done by immunoturbidimetry, though hs-CRP assessment can be done by different methods such as immunonephelometry, immunoturbidimetry, ELISA, mass spectrometry, etc. Immunoturbidimetry assays with a detection time of <1 hr have excellent sensitivity (better than 2.8 ꭒg/l) [27] and are a simple, fast, low-cost and reliable method.

When evaluated for CH indices, NLR was within the reference range at baseline in both groups, unlike the finding of the previous study [10]. SII and PLR levels were higher in CAD with AP group at baseline than in CAD without AP group, though the difference was statistically nonsignificant. Also, reduction in SII, MLR and PLR after six months of NSRCT was significant in CAD with AP group patients, though no significant reduction was observed at 12 months. Further studies with larger sample sizes are required to evaluate the effect of this marker on CAD with AP patients.

TC ≥200, TGs ≥150, and LDL ≥130 were considered to be indicators of dyslipidemia, and raised levels of LDL cholesterol are a risk factor for the development of atherosclerosis [9]. Statins are recommended as the first line of treatment for lowering the risk of CVD in high-risk individuals with hyperlipidemia. In our study, in the test group, 60% and in the control group, 57% patients were on statins at baseline. Production of IL-6 is interrupted by statins suppressing the stimulatory effect of IL-6 on the generation of CRP, thereby reducing CRP levels [28]. Thus, further studies are required specifically in statin users and statin non-users in CVD patients.

PAI score and lesion size reduced significantly at six and 12 months. Further, 88.6% healing in (PAI ≤ 2) after NSRCT highlights the efficacy of good-quality NSRCT in the reduction of AP. Also, the use of statins in 60% of cases at 12 months could have also been attributed to high healing in CAD with AP group [29]. Further studies are required to estimate the effect of statins on healing in CVD patients.

The presence of confounders is one of the important drawbacks in assessing the relationship between AP and inflammatory biomarkers. To eliminate potential confounders, age, gender and BMI-matched controls were enrolled, and CVD patients with other comorbidities such as liver, renal, and metabolic disease were excluded. Strict inclusion criteria were employed to control potential oral confounders, including the exclusion of patients with ≥ Stage 2 periodontitis [26] and conventional cardiovascular risk factors, like BMI and smokers, because they may have an impact on inflammatory biomarkers and the outcome of NSRCT [19]. Throughout the study, none of the patients enrolled reported any notable changes in their habits or medical history. All patients were consulted before other interventions that could alter hs-CRP levels.

However, in our study, there were certain limitations. Although strict criteria for participation in the study reduced the possibility of affecting results, still, a variety of conditions, including sleeplessness and malnutrition, can have an impact on hs-CRP levels, which are difficult to control. For ethical reasons, additional biochemical tests were not conducted in the control group during the follow-up period. Comparison of the levels of systemic biomarkers in both groups at follow-ups could have truly shown the impact of non-surgical root canal treatment with control for the confounder of medical treatments/medications. Aging is considered a risk factor for CVD [30]. It is more prevalent in people over 65 years of age than in younger people. Age as a confounding factor was not controlled. The small number of female (n=4) patients included prevented gender variance from being analyzed.

## Conclusions

Significantly higher systemic inflammatory burden (SIB) as assessed by systemic inflammatory biomarkers was observed in patients with CAD with AP than in CAD without AP controls, suggesting that AP contributed to increased systemic inflammation. SIB reduced significantly after NSRCT, highlighting the efficacy of NSRCT in reducing systemic inflammatory burden and, hence, cardiovascular risk. More evidence, represented preferably by randomized clinical trials performed on a large scale, is required to obtain proven results regarding the non-surgical root canal treatment and cardiovascular disease relationship.
